# Impact of a Mediterranean Dietary Pattern and Its Components on Cardiovascular Risk Factors, Glucose Control, and Body Weight in People with Type 2 Diabetes: A Real-Life Study

**DOI:** 10.3390/nu10081067

**Published:** 2018-08-10

**Authors:** Marilena Vitale, Maria Masulli, Ilaria Calabrese, Angela Albarosa Rivellese, Enzo Bonora, Stefano Signorini, Gabriele Perriello, Sebastiano Squatrito, Raffaella Buzzetti, Giovanni Sartore, Anna Carla Babini, Giovanna Gregori, Carla Giordano, Gennaro Clemente, Sara Grioni, Pasquale Dolce, Gabriele Riccardi, Olga Vaccaro, on behalf of the TOSCA.IT Study Group

**Affiliations:** 1 Department of Clinical Medicine and Surgery, Federico II University of Naples, 80131 Naples, Italy; marilena.vitale@unina.it (M.V.); maria.masulli@unina.it (M.M.); ilariacalabrese@live.it (I.C.); rivelles@unina.it (A.A.R.); riccardi@unina.it (G.R.); 2 Division of Endocrinology, Diabetes and Metabolism, University and Hospital Trust of Verona, 37134 Verona, Italy; enzo.bonora@univr.it; 3 University Department Laboratory Medicine, Hospital of Desio, 20832 Monza, Italy; s.signorini@asst-monza.it; 4 Endocrinology and Metabolism, University of Perugia, 06126, Perugia, Italy; gabriele.perriello@gmail.com; 5 Diabetes Unit, University Hospital Garibaldi-Nesima of Catania, 95122 Catania, Italy; squatrit@unict.it; 6 Department of Experimental Medicine, Sapienza University, 04100 Rome, Italy; raffaella.buzzetti@uniroma1.it; 7 Department of Medicine, University of Padua, 35100 Padova, Italy; g.sartore@unipd.it; 8 Medical Division, Rimini Hospital, 47900 Rimini, Italy; acbabini@auslrn.net; 9 Diabetes Unit, Azienda Sanitaria Toscana Nord-Ovest, Massa Carrara, 54100 Massa Carrara, Italy; g.gregori@usl1.toscana.it; 10 Section of Endocrinology, Diabetology and Metabolic Diseases, University of Palermo, 90127 Palermo, Italy; carlagiordano53@gmail.com; 11 Institute for Research on Population and Social Policies—National Research Council, 84084 Fisciano, Italy; gennaro.clemente@cnr.it; 12 Unità di Epidemiologia e Prevenzione, Fondazione IRCCS, Istituto Nazionale Tumori, 20133 Milano, Italy; sara.grioni@istitutotumori.mi.it; 13 Department of Public Health, Federico II University of Naples, 80131 Naples, Italy; pasquale.dolce@unina.it

**Keywords:** Mediterranean diet, diabetes, cardiovascular risk, glucose control, plasma lipids, relative Mediterranean diet score

## Abstract

This study evaluates the relation of a Mediterranean dietary pattern and its individual components with the cardiovascular risk factors profile, plasma glucose and body mass index (BMI) in people with type 2 diabetes. We studied 2568 participants at 57 diabetes clinics. Diet was assessed with the EPIC (European Prospective Investigation into Cancer and Nutrition) questionnaire, adherence to the Mediterranean diet was evaluated with the relative Mediterranean diet score (rMED). A high compared to a low score was associated with a better quality of diet and a greater adherence to the nutritional recommendations for diabetes. However, even in the group achieving a high score, only a small proportion of participants met the recommendations for fiber and saturated fat (respectively 17% and 30%). Nonetheless, a high score was associated with lower values of plasma lipids, blood pressure, glycated hemoglobin, and BMI. The relationship of the single food items components of the rMED score with the achievement of treatment targets for plasma lipids, blood pressure, glucose, and BMI were also explored. The study findings support the Mediterranean dietary model as a suitable model for type 2 diabetes and the concept that the beneficial health effects of the Mediterranean diet lie primarily in its synergy among various nutrients and foods rather than on any individual component.

## 1. Introduction

Diet remains the cornerstone of effective type 2 diabetes management; the aim of promoting nutritional changes in people with diabetes is to optimize metabolic control and overall health. Nutritional recommendations have been issued by several scientific societies to support clinicians in the choice of the most suitable dietary intervention(s) in people with diabetes [1]. However, adherence to these recommendations in real life clinical practice is generally poor [2,3,4] and partly reflects the wider problem of the overabundance of saturated fat and refined cereals in the western diet [5]. Furthermore, nutritional recommendations are based on nutrients, which might hamper patients’ understanding and compliance. Last but not least, nutritional recommendations have been criticized as being scarcely based on evidence, and there is debate in the literature regarding the optimal dietary macronutrient composition of the diet in people with type 2 diabetes under energy balanced conditions [1,6,7].

In the last decades, human nutrition science has shifted from a reductionist approach focused on specific nutrients to a broader view emphasizing the concepts of overall dietary quality and patterns that promote metabolic health [8]. This paradigm change is supported by convincing evidence that food exposure is complex and its impact on health is influenced not only by single nutrients, but also by their interplay and by the interactions of the bioactive non-nutrients present in food (i.e., fiber, antioxidants, minerals, etc.). Therefore, the relationship between nutrition and health may not be fully appreciated unless evaluated within the context of the whole diet. 

The Mediterranean diet is among the most widely studied dietary patterns. The traditional Mediterranean diet is characterized by the consumption of whole grains, legumes, fruits, vegetables, nuts, fish and olive oil, wine in moderation, and a moderate intake of meat, dairy products, processed foods and sweets. The Mediterranean dietary pattern is also an important source of vitamins, minerals, antioxidants, mono- and poly-unsaturated fatty acids, and fiber—all of which provide a wide range of health benefits. There is abundant evidence of its health benefits [9,10,11,12]; in addition, this type of diet has also a great potential for long-term adherence and sustainability [13]. However, data in populations with diabetes are scant; available information is mostly restricted to the experimental setting of controlled trials whereas little is known on the impact of a Mediterranean like dietary pattern on metabolic outcomes in real life clinical practice [14,15]. Furthermore, Mediterranean diet is a broad term used to describe the traditional food choices of people living around the Mediterranean basin, but there is remarkably little information on the protective/detrimental health impact of specific food groups. In particular, it is unclear whether the beneficial health effects of the Mediterranean diet are due to the diet as a whole or are driven by key food/food components that could also be provided as supplements.

Against this background, the aims of the study were to analyze the food and nutrient intake of a large cohort of people with type 2 diabetes in real-life clinical practice, to explore the impact of a Mediterranean-like dietary pattern on major cardiovascular risk factors, glucose control and body weight, and identify whether and to what extent the beneficial effect of the Mediterranean diet are driven by some food items/components which may be particularly beneficial for people with type 2 diabetes.

## 2. Materials and Methods

### 2.1. Study Population

To explore the study questions, we used data collected within the framework of the TOSCA.IT study—a randomized controlled trial (NCT00700856) designed to compare the effects of a sulfonylurea or pioglitazone, in add-on to metformin, on cardiovascular events in people with type 2 diabetes inadequately controlled with metformin monotherapy. Details on inclusion and exclusion criteria are reported elsewhere [16,17]. Briefly men and women with type 2 diabetes, aged 50–75 years, with glycated hemoglobin 7.0–9.0%, were recruited in 57 centers distributed throughout Italy. People with impaired renal function (serum creatinine ≥ 1.5 mg/dL), a cardiovascular event in the previous six months, and conditions other than diabetes requiring special dietary treatment were excluded from the study. The study protocol was approved by the Ethics Review Board of the Coordinating Center and of each participating center, and written informed consent was obtained from all participants before entering the study. For the purposes of this study, only baseline data, collected prior to the randomization to the study treatments, were used. The present analyses include 2568 men and women with a complete data set.

### 2.2. Measurements

Body weight, height, waist and hip circumference were measured with standard procedures, body mass index (BMI) was calculated as weight (kg)/height (m^2^). Sitting blood pressure was measured according to a standard protocol. Blood samples were obtained in the morning after an overnight fast, all biochemical analyses were performed in a central laboratory. Total and HDL cholesterol, triglycerides and high sensitivity C-reactive protein (CRP) were measured by standard methods. LDL cholesterol was calculated according to the Friedewald equation only for triglyceride values <400 mg/dL. Glycated hemoglobin (HbA1c) was measured with high liquid performance chromatography standardized according to IFCC. 

### 2.3. Evaluation of Eating Habits

Eating habits were assessed with the European Prospective Investigation into Cancer and Nutrition (EPIC) questionnaire, a validated method frequently used in large epidemiological studies [18,19]; details have been given elsewhere [3,6]. Briefly, the questionnaire contained 248 items on 188 different foods including the type of fat used as condiment or added after cooking. People were asked to indicate the absolute frequency of consumption of each item (per day, week, month or year), and the quantity of the food consumed by selection of pictures showing a small, medium and large portion size, with additional quantifiers (e.g., “smaller than the small portion” or “between the small and medium portion”, etc.). Incomplete questionnaires and questionnaires with implausible data (i.e., energy intake less than 800 or greater than 5000 kcal/day) were excluded from the analyses. A specific software (Nutrition Analysis of food frequency questionnaire—FFQ), developed by the Epidemiology and Prevention Unit, Fondazione IRCCS, Istituto Nazionale dei Tumori, Milan, was used to convert dietary data from the questionnaire into average daily amounts of foods (g/day) [18,19]. Nutrition analysis of FFQ was linked to the Italian Food Composition Tables (FCTs) for nutrients and energy assessment [20,21]. The intake of polyphenols was evaluated using the USDA database [22] in combination with the Phenol-Explorer® database [23] to enable the examination of the polyphenol content of as many foods as possible. Details have been given elsewhere [24,25].

### 2.4. Adherence to the Mediterranean Diet

The adherence to the Mediterranean dietary model was evaluated with the rMED score (relative Mediterranean diet score), a variation of the original Mediterranean diet score, proposed by Buckland [26] and based on the intake of 9 key food groups: fruits, vegetables, legumes, cereals, fish, olive oil, meat and meat products, dairy products, and alcohol. The consumption of each food group (except alcohol) was measured as grams per 1000 kcal/day to adjust for energy density and divided into tertiles. A score of 0, 1, or 2 was assigned to the first, second, or third tertile of intake, assigning a positive score for high intakes for the 6 food groups fitting the Mediterranean model: fruit (including nuts and seeds but excluding fruit juices), vegetables (excluding potatoes), legumes, cereals (including whole-grain and refined flour, pasta, rice, other grains, and bread), fish and seafood, olive oil. The scoring was reversed for meat (including fresh and processed meat) and dairy products (including high- and low-fat milk, cheese, cream desserts, and dairy and nondairy creams), assigning a positive score for lower intakes. Alcohol was scored as a dichotomous variable as in prior studies: two points were assigned for moderate consumption—defined as 5–25 g/day for women and 10–50 g/day for men—and 0 points were assigned for a consumption above or below the sex-specific range. For each participant a total score was calculated by summing the scores obtained for each of the 9 food groups. Values for the rMED score ranged from 0 to 18; based on this score, three groups with low (score 0–6), intermediate (score 7–10) or high (score 11–18) were created [26]. The rMED score was selected among others for this study as it excludes sweetened beverages and potatoes which are foods restricted in people with diabetes.

### 2.5. Statistical Analysis

Data are shown as mean and standard deviation (M ± SD) or number and proportions, as appropriate. The analysis of variance (ANOVA) with linear term and the *post-hoc* test adjusted for multiple comparisons (Bonferroni test) were used to test for the differences in the composition of the habitual diet, blood pressure and metabolic parameters across categories of adherence to the Mediterranean Diet and between the highest vs. lowest rMED score, respectively. The λ^2^ test was used to compare proportions. A binary logistic regression analysis was performed to evaluate the association of total rMED score and single food groups components of the rMED score with the achievement of treatment targets for the major cardiovascular risk factors (i.e., LDL cholesterol, triglycerides, HDL cholesterol, blood pressure), measures of glucose control—glycated hemoglobin—and BMI. A *p*-value < 0.05 (two-tailed) was considered statistically significant. All analyses were conducted with the SPSS Statistics software for Windows (version 20.0; SPSS Inc., Chicago, IL, USA).

## 3. Results

The study population consists of 1534 males and 1034 females with mean age 62.1 ± 6.5 years and BMI 30.3 ± 4.4 kg/m^2^. Table 1 shows the general features of the study participants according to the rMED score groups. A high adherence score was significantly more frequent among females (*p* = 0.002), older people (*p* = 0.027) and residents of the southern regions (*p* < 0.0001). No relation was found with education, smoking, or marital status.

Table 2 and Table 3 report the average food consumption and nutrient composition of the diet in the three rMED score groups. By definition, people with a high adherence score consumed substantially more fruit, vegetables, legumes, cereals, fish, olive oil, and alcohol, and substantially less meat, and dairy products (Table 2).

Eating a high rMED diet was characterized by a lower energy content, a lower intake of proteins from animal food sources, saturated fat and cholesterol, added sugars, a higher intake of fiber and a lower glycemic index and glycemic load (Table 3). As for micronutrients, a high rMED score was associated with a significantly lower intake of calcium and sodium and a significantly higher intake of total polyphenols (Table 3); no significant difference was detected for potassium intake. 

**Figure 1:**
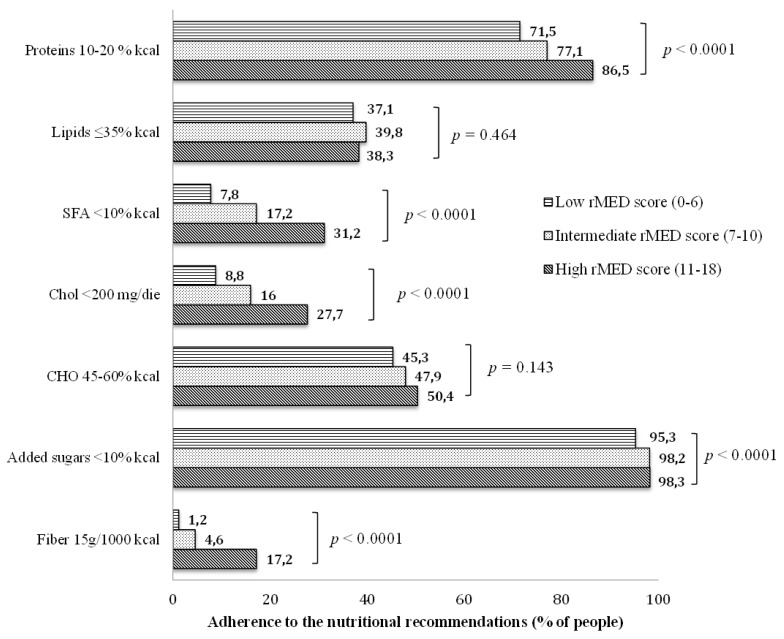
Adherence to the nutritional recommendations for people with diabetes (DNSG [27] and SID [28]) by rMED score. DNSG: Diabetes and Nutrition Study Group; SID: Italian Diabetes Society; SFA: Saturated Fatty Acids; Chol: Cholesterol; CHO: Carbohydrates.

We also evaluated the adherence to the current nutritional recommendation for people with diabetes in the participants with low, intermediate or high rMED score (Figure 1). Increasing rMED score values were associated with higher adherence to the nutritional recommendations. Interestingly, whereas the adherence to the nutritional recommendations for added sugar and carbohydrates was good in all three groups, the adherence to the recommendations for fiber and saturated fat remained low even in the high rMED score group. In this group, the proportion of adherence was respectively 31% for saturated fat and 17% for fiber, which is significantly higher than in the low rMED score group, although still far from optimal. 

With regard to the cardiovascular risk factors profile, a high versus low rMED score was associated with a more favorable plasma lipid profile—i.e., lower LDL cholesterol (101.5 ± 31.2 vs. 105.1 ± 31.9 mg/dL, *p* = 0.035) and triglycerides (146.7 ± 71.0 vs. 156.2 ± 78.6 mg/dL, *p* = 0.040), and higher HDL cholesterol (46.8 ± 12.4 vs. 45.3 ± 11.6 mg/dL, *p* = 0.032), significantly lower blood pressure—systolic (133.3 ± 23.7 vs. 135.3 ± 14.9 mmHg, *p* = 0.045) and diastolic (78.6 ± 8.5 vs. 80.7 ± 8.7 mmHg, *p* < 0.0001)—lower HbA1c (7.63 ± 0.48 vs. 7.69 ± 0.52%, *p* = 0.038), lower BMI (30.0 ± 4.2 vs. 30.6 ± 4.5 kg/m^2^, *p* = 0.020), and lower C-reactive protein (3.12 ± 4.8 vs. 3.79 ± 6.7 mg/L, *p* = 0.029) (Table 4). Of note, the proportion of people on lipid- or blood pressure-lowering drugs was not significantly different across the three groups (Table 4), thus suggesting a significant effect of diet beyond the effect of drugs.

Finally, we explored the association of the rMED score globally and for the single food groups with the achievement of treatment targets for plasma lipids, blood pressure, HbA1c, and BMI (Table 5). The odds of reaching the treatment target for LDL cholesterol increased by 13% per unit increase in the rMED score for fruit and nuts (OR 1.134; CI 1.006–1.277); for triglycerides, there was a significant association with fish consumption (OR 1.128; CI 1.003–1.269), and for HDL cholesterol a significant association was found for fruit and nuts (OR 1.142; CI 1.016–1.283) and alcohol (moderate consumption) (OR 1.206; CI 1.090–1.335). As for systolic blood pressure, the score for fruit and nuts (OR 1.174; CI 1.034–1.333), legumes (OR 1.259; CI 1.106–1.433), cereals (OR 1.133; CI 1.001–1.284), fish (OR 1.146; CI 1.013–1.297) and meat (inverse) (OR 1.170; CI 1.035–1.323) were all significantly associated with achievement of treatment targets; data for diastolic blood pressure were similar (not shown). The score for meat (low consumption) (OR 1.141; CI 1.035–1.258), fish (OR 1.109; CI 1.004–1.225), and alcohol (moderate consumption) (OR 1.183; CI 1.090–1.284) were also significantly associated with likelihood of a BMI below 30 kg/m^2^. For HbA1c, a significant association was found for fish (inverse) (OR 0.888; CI 0.803–0.981) and dairy products (low consumption) (OR 1.154; CI 1.045–1.273).

## 4. Discussion

Several scores have been developed to evaluate the degree of adherence to the Mediterranean Diet, but none has been validated so far for the use in people with diabetes, for whom nutritional therapy and, hence, food choice restrictions and limited consumption of selected food is recommended. The reason we selected the rMED score for this study [26] is because it excludes sweetened beverages and potatoes which are foods restricted in people with diabetes. 

Although not specifically validated for people with diabetes, the rMED score efficiently identified three groups with substantially different eating habits. The study results show that in real-life clinical practice, the dietary habits of people with type 2 diabetes vary significantly with gender, age, and area of residence. In particular, females, older people, and residents of the southern regions tend to adhere more to a Mediterranean eating pattern. 

The habitual diet of people with a high rMED score, as compared to that of people with a low score, was characterized by a lower energy intake, a lower intake of saturated fat and cholesterol, a higher intake of fish, vegetable proteins and fiber; glycemic index and glycemic load were also significantly lower, as was the intake of sodium and calcium, whereas the intake total polyphenols was significantly higher. On the overall, this group had a significantly less atherogenic and less proinflammatory diet. Nevertheless, even in the group with the highest score, the intake of fiber and saturated fat remained respectively lower and higher than recommended by the European and Italian nutritional guidelines for people with diabetes [27,28]. 

A low consumption of fiber and a relatively high intake of saturated fat have been reported by other studies in type 1 and type 2 diabetes [2,3,4] and most likely reflect the wider problem of a progressive shifting towards more western dietary models in all cultures, including countries with strong Mediterranean roots like Italy [29,30]. This notwithstanding, a high rMED score is associated with a more favorable cardiovascular risk factors profile, lower BMI, lower HbA1c, and lower subclinical inflammation. The magnitude of the differences between the high and low rMED score group may seem trivial, but if translated at the population level, may considerably impact on the absolute cardiovascular risk of the study population. Based on prior observational and intervention studies exploring the impact of the modification of major cardiovascular risk factors on the absolute cardiovascular risk [31,32,33,34,35], it can be estimated that combining the differences between the high and low rMED score groups in LDL cholesterol, triglycerides, HDL cholesterol, blood pressure, and HbA1c could result in a 21% reduction of the estimated absolute cardiovascular risk. Thus, emphasizing that the individual effects of the Mediterranean diet are small but taken as a whole the effects are large.

To our knowledge, this is one of the very few studies exploring the impact of a Mediterranean-like dietary pattern on glucose control and major cardiovascular risk factors in people with type 2 diabetes in real-life conditions. Most prior evidence on the beneficial effects of a Mediterranean diet model in people with diabetes comes from intervention trials, often of short duration, some of which have used food supplements [14,15,36]. The results of this study are in line with observational studies conducted in people without diabetes, and with a recent observational study conducted in a community-based sample of people with type 2 diabetes showing a significant reduction of all cause and cardiovascular deaths in patients who adhered most to the Mediterranean diet [37]. However, the lack of data on intermediate outcomes in this study does not allow comparisons with our findings. In addition, there is no standard definition for the Mediterranean diet, and adherence scores are based on population specific cut-off values for food consumption; this makes them poorly reproducible when utilized in different population groups, and further limits comparison between different studies.

We also explored the relation between scores of each individual food group component of the Mediterranean diet and the achievement of treatment targets for individual risk factors. Based on these analyses, a differential effect of single food groups was observed with regard to different risk factors (i.e., increasing the scores for fruit and vegetables significantly improved the probability of reaching the treatment target for LDL cholesterol; increasing the consumption of fish significantly improve the likelihood of reaching the target for triglycerides; the scores for legumes and vegetables were the main drivers for the achievement of treatment targets for blood pressure, etc.). All together, these data point to the conclusion that the beneficial health effects of the Mediterranean diet are largely due to the overall diet rather than being driven by single components, as different food items target different risk factors. 

The major study strengths rely on the large sample size, the selection of a study population representative of real-life clinical practice, the standardized collection of nutritional and clinical data and the centralized biochemical measurements. Among the study limitations, we acknowledge the cross-sectional design and the use of intermediate endpoints. In addition, the dietary data were collected only once and could be prone to recall bias and seasonal variation, which might, however, bias the findings towards null, thus leading to the underestimation of the effect size. Finally, the extensive use of hypolipidemic and antihypertensive drugs could have partly offset the quantitative effect of nutritional factors. In this regard, the appreciation of the impact of dietary adherence in the face of pharmacological treatment was even more relevant.

## 5. Conclusions

In conclusion, a dietary pattern mimicking the Mediterranean model in people with type 2 diabetes is associated with more favorable cardiovascular risk factors profile, better glucose control and lower BMI and it is therefore a valid and sustainable nutritional strategy for people with diabetes in real-life clinical practice. However, a high rMED score in this population does not guarantee an ideal adherence to the nutritional recommendations for the management of diabetes, in fact, the intake of saturated fat and fiber in the highest rMED score group remain respectively higher and lower than recommended. These findings together with available evidence from other observational and intervention studies emphasize the need to reinforce the importance of higher fiber, low glycemic index foods such as legumes, fruit and vegetables, wholegrain cereals, and the substitution of monounsaturated for saturated fat sources, in energy balanced conditions, in people with diabetes.

Large-scale primary prevention trials focused on dietary patterns and cardiovascular disease risk in people with diabetes are unlikely to be undertaken; hence, observational findings such as these represent an important basis for dietary recommendations, government programs, and negotiations with industry to help people make healthy food choices. 

## Figures and Tables

**Table 1:** Characteristics of the study participants by rMED score groups.

	rMED Score Groups			*p*-Value
	Low (Score 0–6) (*n* = 834)	Intermediate (Score 7–10) (*n* = 1029)	High (Score 11–18) (*n* = 705)	
**Age (years)**				
<60 years (%)	316 (37.9)	355 (34.5)	221 (31.3)	0.027
≥60 years (%)	518 (62.1)	674 (65.5)	484 (68.7)	
**Sex**				
Men (%)	537 (64.4)	601 (58.4)	396 (56.2)	0.002
Women (%)	297 (35.6)	428 (41.6)	309 (43.8)	
**Geographical Area**				
North (%)	369 (44.2)	349 (33.9)	175 (24.8)	
Centre (%)	219 (26.3)	287 (27.9)	171 (24.3)	<0.0001
South (%)	246 (29.5)	393 (38.2)	359 (50.9)	
**Education**				
Secondary/University (%)	261 (31.3)	326 (31.7)	232 (33.0)	0.607
None/Primary (%)	573 (68.7)	703 (68.3)	472 (67.0)	
**Smoking status ^1^**				
Never smoker (%)	388 (46.5)	510 (49.6)	341 (48.4)	
Current Smoker (%)	152 (18.2)	182 (17.7)	110 (15.6)	0.376
Former Smoker (%)	294 (35.3)	337 (32.8)	254 (36.0)	
**Marital status**				
Married (%)	697 (83.6)	868 (84.3)	615 (87.2)	0.112
Single or widowed (%)	137 (16.4)	161 (15.6)	90 (12.8)	

Data are expressed as number and percentage. Subjects are classified as “current smokers” if they smoke ≥5 cigarettes/day, and “former smokers” if they had smoked in the past and had stopped smoking for at least 1 year.

**Table 2:** Consumption of food groups (expressed as g/1000 kcal/day) by rMED score groups.

rMED Score Groups				
Food Item	Low (Score 0–6) (*n* = 834)	Intermediate (Score 7–10) (*n* = 1029)	High (Score 11–18) (*n* = 705)	*p*-Value for Trend
Fruit & Nuts	125.8 ± 79.6	161.2 ± 93.2	200.7 ± 84 *	<0.0001
Vegetables	66.7 ± 36.1	96.9 ± 46.2	124 ± 44.6 *	<0.0001
Legumes	8.2 ± 7.2	13.5 ± 10.5	19.3 ± 12.1 *	<0.0001
Cereals	90.3 ± 35.5	95.8 ± 36.7	95.6 ± 32.6 *	0.033
Fish	16.9 ± 13.1	22.8 ± 16.4	28.9 ± 18.1 *	<0.0001
Olive oil	9.4 ± 4.7	13 ± 5.6	16.4 ± 5.6 *	<0.0001
Meat	76.4 ± 28.2	68 ± 27.8	58.7 ± 24.5 *	<0.0001
Dairy products	24.8 ± 14.5	20.3 ± 12.2	14.8 ± 10.2 *	<0.0001
Alcohol	0.27 ± 0.44	0.41 ± 0.50	0.50 ± 0.50 *	<0.0001

Data are expressed as mean ± standard deviation; * *p* < 0.001 vs. low score, *post-hoc* test adjusted for multiple comparisons (Bonferroni Test).

**Table 3:** Energy and nutrient composition of the diet by rMED score groups.

	rMED Score Groups			*p*-Value for Trend
	Low (Score 0–6) (*n* = 834)	Intermediate (Score 7–10) (*n* = 1029)	High (Score 11–18) (*n* = 705)	
Total Energy (kcal/day)	2093 ± 773	1890 ± 638	1718 ± 561 *	<0.0001
Proteins (% TE)	18.6 ± 2.5	18.3 ± 2.6	17.7 ± 2.3 *	<0.0001
Animal sources (% TE)	13.4 ± 3	12.55 ± 3.2	11.6 ± 2.8 *	<0.0001
Vegetable sources (% TE)	5.2 ± 1.1	5.7 ± 1.1	6.1 ± 1 *	<0.0001
Lipids (% TE)	37.0 ± 5.8	36.5 ± 6.3	36.5 ± 5.6	0.166
SFA (% TE)	13.4 ± 2.5	12.1 ± 2.3	10.9 ± 2 *	<0.0001
MUFA (% TE)	16.9 ± 3.3	17.9 ± 3.9	19.0 ± 3.7 *	<0.0001
PUFA (% TE)	4.3 ± 1.1	4.4 ± 1.2	4.5 ± 1.0 *	0.034
Cholesterol (mg/die)	379 ± 162	325 ± 133	272 ± 111 *	<0.0001
Carbohydrates (% TE)	44.3 ± 7.2	45.1 ± 7.6	45.8 ± 6.5 *	<0.0001
Added sugars (% TE)	3.0 ± 3.7	2.2 ± 3.0	2.0 ± 2.7 *	<0.0001
Fiber (g/1000 kcal/day)	8.8 ± 2.0	10.8 ± 2.3	12.8 ± 2.4 *	<0.0001
Glycaemic Index	52.7 ± 3.7	51.8 ± 3.5	51.3 ± 3.2*	<0.0001
Glycaemic load	143.4 ± 68.2	113.8 ± 46.8	98.9 ± 37.5 *	<0.0001
Alcohol (g/die)	12.9 ± 20.5	10.2 ± 13.5	10.4 ± 12.2 *	<0.0001
Calcium (mg)	1007 ± 476	880 ± 370	759 ± 333 *	<.0001
Sodium (mg)	2453 ± 1132	2077 ± 938	1758 ± 752 *	<0.0001
Potassium (mg)	3045 ± 1087	3045 ± 946	3072 ± 984	0.832
Total Polyphenols (mg)	653 ± 317	674 ± 289	733 ± 280 *	<0.0001

Data are expressed as mean ± standard deviation; * *p* < 0.001 vs. low score, *Post-hoc* test adjusted for multiple comparisons (Bonferroni Test). TE: Total Energy; SFA: Saturated fatty acids; MUFA: Monounsaturated fatty acids; PUFA: Polyunsaturated fatty acids.

**Table 4:** Cardiovascular risk factors profile by rMED score groups.

	rMED Score Groups			*p*-Value for Trend
	Low (Score 0–6) (*n* = 834)	Intermediate(Score 7–10) (*n* = 1029)	High (Score 11–18) (*n* = 705)	
BMI (kg/m^2^)	30.6 ± 4.5	30.1 ± 4.4	30.0 ± 4.2 *	0.020
HbA1c (%)	7.69 ± 0.52	7.67 ± 0.49	7.63 ± 0.48 *	0.038
LDL cholesterol (mg/dL)	105.1 ± 31.9	101.8 ± 30.8	101.5 ± 31.2 *	0.035
HDL cholesterol (mg/dL)	45.3 ± 11.6	45.8 ± 11.4	46.8 ± 12.4 *	0.032
Triglycerides (mg/dL)	156.2 ± 78.6	150.2 ± 73.9	146.7 ± 71.0 *	0.040
Systolic blood pressure (mmHg)	135.3 ± 14.9	133.5 ± 14.4	133.3 ± 23.7 *	0.045
Diastolic blood pressure (mmHg)	80.7 ± 8.7	79.3 ± 8.4	78.6 ± 8.5 *	<0.0001
C-reactive protein ^1^ (mg/L)	3.79 ± 6.7	3.23 ± 4.7	3.12 ± 4.8 *	0.029
People on blood pressure lowering drugs (%)	73.7	71.9	68.4	0.063
People on lipid lowering drugs (%)	65.1	66.6	67.0	0.702

M ± SD or %. * *p* < 0.05 vs. low score. *Post-hoc* test adjusted for multiple comparisons (Bonferroni Test). ^1^ Excluding subjects with C-reactive protein value >100 mg/L.

**Table 5:** Odds ratio (95% CI) for the achievement of treatment target for LDL cholesterol, triglycerides, HDL-cholesterol, systolic blood pressure, BMI, and HbA1c associated to one-point increase of the total rMED score and of the score for each food item component of the score.

	Odd Ratio (95% CI)					
	LDL-Chol <100 mg/dL	Triglycerides <150 mg/dL	HDL-Chol >40 M or 50 F mg/dL	Systolic BP <130 mmHg	HbA1c <7.5%	BMI <30 kg/m^2^
Total rMED Score	1.119 (1.002–1.250) *	1.128 (1.036–1.228) *	1.150 (1.006–1.315) *	1.305 (1.100–1.548) *	1.087 (1.001–1.180) *	1.097 (1.005–1.197) *
Mediterranean food item						
Fruits and Nuts	1.134 (1.006–1.277) *	1.024 (0.908–1.155)	1.142 (1.016–1.283) *	1.174 (1.034–1.333) *	1.041 (0.941–1.152)	0.997 (0.902–1.102)
Vegetables	0.979 (0.848–1.132)	1.017 (0.879–1.177)	0.948 (0.821–1.094)	1.074 (0.921–1.252)	1.056 (0.928–1.202)	0.901 (0.793–1.024)
Legumes	1.015 (0.898–1.146)	1.004 (0.888–1.136)	1.013 (0.897–1.144)	1.259 (1.106–1.433) *	1.057 (0.954–1.171)	0.981 (0.874–1.010)
Cereals	0.953 (0.848–1.070)	1.009 (0.897–1.135)	0.917 (0.816–1.030)	1.133 (1.001–1.284) *	0.924 (0.834–1.024)	1.049 (0.947–1.161)
Meat and meat products (low intake)	1.051 (0.936–1.180)	1.003 (0.892–1.127)	1.020 (0.909–1.145)	1.170 (1.035–1.323) *	1.082 (0.981–1.194)	1.141 (1.035–1.258) *
Fish	1.073 (0.955–1.205)	1.128 (1.003–1.269) *	0.978 (0.871–1.098)	1.146 (1.013–1.297) *	0.888 (0.803–0.981) *	1.109 (1.004–1.225) *
Dairy products (low intake)	1.036 (0.922–1.165)	0.958 (0.851–1.078)	1.006 (0.895–1.131)	1.044 (0.922–1.182)	1.154 (1.045–1.273) *	1.005 (0.911–1.108)
Olive oil	0.928 (0.806–1.068)	1.053 (0.914–1.214)	0.975 (0.847–1.122)	0.982 (0.845–1.140)	0.974 (0.858–1.107)	1.066 (0.939–1.210)
Moderate alcohol consumption	1.052 (0.951–1.165)	1.075 (0.970–1.191)	1.206 (1.090–1.335) *	1.061 (0.952–1.182)	1.019 (0.939–1.106)	1.183 (1.090–1.284) *

* *p* < 0.05.

## Supplementary Materials

The following are available online at http://www.mdpi.com/2072-6643/10/8/1067/s1, complete list of collaborators, members of the TOSCA.IT Study Group (surname is reported in bold).
