# Supplementary material for: Impact of a Mediterranean Dietary Pattern and Its Components on Cardiovascular Risk Factors, Glucose Control, and Body Weight in People with Type 2 Diabetes: A Real-Life Study
Source: Nutrients. 2018 Aug 10;10(8):1067. doi: 10.3390/nu10081067 (PMC6115857; doi:10.3390/nu10081067)
Supplement: Supplementary file 1 [file nutrients-10-01067-s001.pdf]

**Complete list of collaborators, members of the TOSCA.IT Study Group (surname is reported in bold)**

Olga **Vaccaro**, Maria **Masulli**, Antonio **Nicolucci**, Aldo Pietro **Maggioni**, Paolo **Mocarelli**, Giuseppe **Lucisano**, Michele **Sacco**, Stefano **Signorini**, Fabrizio **Cappellini**, Gabriele **Riccardi**, Massimo **Boemi**†, Federica **D'Angelo**, Roberto **Giansanti**, Laura **Tanase**, Luigi **Lanari**, Ivano **Testa**, Lucia **Ricci**, Francesca **Pancani**, Anna **Ranchelli**, Paolo **Vagheggi**, Alessia **Scatona**, Lucia **Fontana**, Francesco **Giorgino**, Luigi **Laviola**, Lucia **Tarantino**, Claudia **Ippolito**, Vittoria **Gigantelli**, Mariangela **Manicone**, Eleonora **Conte**, Roberto **Trevisan**, Cristiana **Scaranna**, Rossella **Rota**, Anna **Corsi**, Alessandro R. **Dodesini**, Giulio **Marchesini Reggiani**, Luca **Montesi**, Natalia **Mazzella**, Gabriele **Forlani**, Chiara **Caselli**, Raffaella **Di Luzio**, Arianna **Mazzotti**, Antimo **Aiello**, Angelina **Barrea**, Fiorentina **D'Amico,** Sebastiano **Squatrito**, Tiziana **Sinagra**, Sara **Longhitano**, Vanessa **Tropea**, Maria **Sparti**, Salvatore **Italia**, Enrico **Lisi**, Giuseppe **Grasso**, Federica **Insalaco**, Agostino **Gnasso**, Claudio **Carallo**, Caterina **Scicchitano**, Maria Serena **De Franceschi**, Costanza **Santini**, Giovanni **Calbucci**, Raffaella **Ripani**, Laura **Corsi**, Giacomo **Cuneo**, Simona **Corsi**, Carlo B. **Giorda**, Francesco **Romeo**, Annalisa **Lesina**, Marco **Comoglio**, Caterina **Bonetto**, Anna **Robusto**, Elisa **Nada**, Vincenzo **Asprino**, Rosa **Cetraro**, Michelina **Impieri**, Giuseppe **Lucchese**, Giovanna **Donnarumma**, Biagio **Tizio**, Gennaro **Clemente**, Lazzaro **Lenza**, Pia **Paraggio**, Franco **Tomasi**, Chiara **Zamboni**, Nicoletta **Dozio**, Egle **Scalambra**, Edoardo **Mannucci**, Caterina **Lamanna**, Mauro **Cignarelli**†, Olga **La Macchia**, Stefania **Fariello**, Maria Rosaria **Sorrentino**, Ivano **Franzetti**, Raffaella **Radin**, Renzo **Cordera**, Francesca **Annunziata**, Laura **Affinito Bonabello**, Arianna **Durante**, Mara **Dolcino**, Fiorenza **Gallo**, Chiara **Mazzucchelli**, Anna **Aleo**, Pierluigi **Melga**, Lucia **Briatore**, Davide **Maggi**, Daniela **Storace**, Francesca **Cecoli**, Daniela **Antenucci**, Ercole **D'Ugo**, Mario **Pupillo**, Maria Pompea Antonia **Baldassarre**, Anita **Minnucci**, Angelo **De Luca**, Antonella **Zugaro**, Livia **Santarelli**, Angela **Bosco**, Vittorio **Petrella**, Grazia Giovanna **La Verghetta**, Rossella **Iannarelli**, Antonella **De Gregorio**, Settimio **D'Andrea**, Anna Elisa **Giuliani**, Lorella **Polidoro**, Alessandra **Sperandio**, Filomena **Sciarretta**, Alfonso **Pezzella**, Raffaella **Buzzetti**, Angela **Carlone**, Stella **Potenziani**, Chiara **Venditti**, Chiara **Foffi**, Salvatore **Carbone**, Laura **Cipolloni**, Chiara **Moretti**, Gaetano **Leto**, Rosalia **Serra**, Francesca **Petrachi**, Isabella **Romano**, Graziano **Di Cianni**, Emilia **Lacaria**, Laura **Russo**, Chiara **Goretti**, Claudia **Sannin**o, Giovanna **Gregori**, Maria **Dolci**, Laura **Bruselli**, Mary L. **Mori**, Fabio **Baccetti**, Maria **Del Freo**, Antonino **Di Benedetto**, Domenico **Cucinotta**, Loretta **Giunta**, Maria Concetta **Ruffo**, Desiree **Cannizzaro**, Basilio **Pintaudi**, Giovanni **Perrone**, Pietro **Pata**, Francesco **Ragonese**, Gabriele **Lettina**, Teresa **Mancuso**, Aldo **Coppolino**, Pier Marco **Piatti**, Lucilla **Monti**, Michela **Stuccillo**, Pietro **Lucotti**, Manuela **Setola**, Giulia Valentina **Crippa**, Cinzia **Loi**, Matteo **Oldani**, Maria Luisa **Bottalico**, Beatrice **Pellegata**, Matteo **Bonomo**, Laura Silvia Maria **Menicatti**, Veronica **Resi**, Federico **Bertuzzi**, Eugenia Olga **Disoteo**, Gianluigi **Pizzi**, Angela Albarosa **Rivellese**, Giovanni **Annuzzi**, Brunella **Capaldo**, Rossella **Nappo**, Stefania Michela **Auciello**, Anna Amelia **Turco**, Lucia **Costagliola**, Ciro **Iovine**, Giuseppina **Della Corte**, Pasquale **Vallefuoco**, Francesca **Nappi**, Marilena **Vitale**, Sara **Cocozza**, Ornella **Ciano**, Elena **Massimino**; Nadia **Garofalo**; Angelo **Avogaro**, Monica **Vedovato**, Gabriella **Guarneri**, Annunziata **Lapolla**, Domenico **Fedele**, Giovanni **Sartore**, Nino Cristiano **Chilelli**, Silvia **Burlina**, Barbara **Bonsembiante**, Carla **Giordano**, Aldo **Galluzzo**†, Vittoria **Torregrossa**, Elisabetta **Dall'Aglio**, Giovanni **Mancastroppa**, Leone **Arsenio**, Federico **Cioni**, Silvana **Caronna**, Matteo **Papi**, Massimiliano **Babini**, Gabriele **Perriello**, Fausto **Santeusanio**, Gioia **Calagreti**, Alessia **Timi**, Alice **Tantucci**, Cecilia **Marino**, Agostino **Consoli**, Federica **Ginestra**, Rosamaria **Di Biagio**, Merilda **Taraborelli**, Stefano **Del Prato**, Roberto **Miccoli**, Cristina **Bianchi**, Monia **Garofolo**, Konstantina Savina **Politi**, Giuseppe **Penno**, Donatella **Zavaroni**, Stefania **Livraga**, Fabio **Calzoni**, Giovanni Luigi Francesco **Mancastroppa**, Roberto **Anichini**, Elisa **Corsini**, Anna **Tedeschi**, Maria Sole **Gaglianò**, Giulio **Ippolito**, Elisabetta **Salutini**, Giuseppe

**Citro**, Maria **Natale**, Vita **Salvatore**, Armando **Zampino**, Rosa **Sinisi**, Maria **Calabrese**, Adolfo **Arcangeli**, Alessia **Zogheri**, Sandra **Guizzotti**, Rossella **Longo**, Paolo **Di Bartolo**, Francesca **Pellicano**, Patrizia **Scolozzi**, Simona **Termine**, Alessandra **Luberto**, Giorgio **Ballardini**, Anna Carla **Babini**, Cristina **Trojani**, Paolo **Mazzuca**, Matteo **Bruglia**, Monica **Ciamei**, Silvia **Genghini**, Chiara **Zannoni**, Giuseppe **Pugliese**, Martina **Vitale**, Graziela **Rangel**, Laura **Salvi**, Alessandra **Zappaterreno**, Samantha **Cordone**, Paola **Simonelli**, Marilla **Meggiorini**, Aurora **Frasheri**, Clelia **Di Pippo**, Cristina **Maglio**, Giulia **Mazzitelli**, Davide **Lauro**, Maria Elena **Rinaldi**, Angelica **Galli**, Maria **Romano**, Paola **D'Angelo**, Sergio **Leotta**†, Concetta **Suraci**, Salvatore **De Cosmo**, Simonetta **Bacci**, Antonio Pio **Palena**, Monica **Mancino**, Maurizio **Rondinelli**, Filippo **Capone**, Elisabetta **Calabretto**, Monica **Bulgheroni**, Loredana **Bucciarelli**, Francesco **Dotta**, Elena **Ceccarelli**, Cecilia **Fondelli**, Clorinda **Santacroce**, Elisa **Guarino**, Laura **Nigi**, Carlo **Lalli**, Maura **Scarponi**, Valeria **Montani**, Paolo **Di Bernardino**, Paola **Romagni**, Katia **Dolcetti**, Emanuela **Cannarsa**, Elisa **Forte**, Lucilla **Tamburo**, Paolo **Fornengo**, Paolo **Cavallo Perin**, Tania **Prinzis**, Chiara **Zucco**, Massimo **Perotta**, Simona **Monsignore**, Francesco **Panero**, Fulvia **Ponzi**, Antonio Carlo **Bossi**, Rita **Carpinteri**, Maria Linda **Casagrande**, Maria Francesca **Coletti**, Annalisa **Balini**, Marcello **Filopanti**, Sara **Madaschi**, Anna **Pulcina**, Franco **Grimaldi**, Laura **Tonutti**, Giorgio **Venturini**, Sandra **Agus**, Stefania **Pagnutti**, Francesca **Guidotti**, Alessandro **Cavarape**, Enzo **Bonora**, Massimo **Cigolini**, Isabella **Pichiri**, Corinna **Brangani**, Giulia **Fainelli**, Elena **Tomasetto**, Giacomo **Zoppini**, Anna **Galletti**, Dominica **Perrone**, Claudio **Capra**, Francesca **Bianchini**, Martina **Ceseri**, Barbara **Di Nardo**, Elisa **Sasso**, Barbara **Bartolomei**, Irina **Suliman**, Gianna **Fabbri**
